# On the measurement uncertainty of microdosimetric quantities using diamond and silicon microdosimeters in carbon‐ion beams

**DOI:** 10.1002/mp.15929

**Published:** 2022-09-09

**Authors:** Cynthia Meouchi, Sandra Barna, Monika Puchalska, Linh T. Tran, Anatoly Rosenfeld, Claudio Verona, Gianluca Verona‐Rinati, Hugo Palmans, Giulio Magrin

**Affiliations:** ^1^ Radiation Physics Technische Universität Wien Vienna Austria; ^2^ Department of Radiation Oncology Medical University of Vienna Vienna Austria; ^3^ Centre for Medical Radiation Physics University of Wollongong Wollongong New South Wales Australia; ^4^ Dipartimento di Ingegneria Industriale Università di Roma Tor Vergata Roma Italy; ^5^ MedAustron Ion Therapy Center Wiener Neustadt Austria; ^6^ National Physical Laboratory Teddington Twickenham UK

**Keywords:** microdosimetry, solid state, uncertainty

## Abstract

**Purpose:**

The purpose of this paper is to compare the response of two different types of solid‐state microdosimeters, that is, silicon and diamond, and their uncertainties. A study of the conversion of silicon microdosimetric spectra to the diamond equivalent for microdosimeters with different geometry of the sensitive volumes is performed, including the use of different stopping power databases.

**Method:**

Diamond and silicon microdosimeters were irradiated under the same conditions, aligned at the same depth in a carbon‐ion beam at the MedAustron ion therapy center. In order to estimate the microdosimetric quantities, the readout electronic linearity was investigated with three different methods, that is, the first being a single linear regression, the second consisting of a double linear regression with a channel transition and last a multiple linear regression by splitting the data into odd and even groups. The uncertainty related to each of these methods was estimated as well. The edge calibration was performed using the intercept with the horizontal axis of the tangent through the inflection point of the Fermi function approximation multi‐channel analyzer spectrum. It was assumed that this point corresponds to the maximum energy difference of particle traversing the sensitive volume (SV) for which the residual range difference in the continuous slowing down approximation is equal to the thickness of the SV of the microdosimeter. Four material conversion methods were explored, the edge method, the density method, the maximum‐deposition energy method and the bin‐by‐bin transformation method. The uncertainties of the microdosimetric quantities resulting from the linearization, the edge calibration and the detectors thickness were also estimated.

**Results:**

It was found that the double linear regression had the lowest uncertainty for both microdosimeters. The propagated standard (*k* = 1) uncertainties on the frequency‐mean lineal energy ${\bar{y}}_{F}$ and the dose‐mean lineal energy ${\bar{y}}_{D}$ values from the marker point, in the spectra, in the plateau were 0.1% and 0.2%, respectively, for the diamond microdosimeter, whilst for the silicon microdosimeter data converted to diamond, the uncertainty was estimated to be 0.1%. In the range corresponding to the 90% of the amplitude of the Bragg Peak at the distal part of the Bragg curve (R_90_) the uncertainty was found to be 0.1%. The uncertainty propagation from the stopping power tables was estimated to be between 5% and 7% depending on the method. The uncertainty on the ${\bar{y}}_{F}$ and ${\bar{y}}_{D}$ coming from the thickness of the detectors varied between 0.3% and 0.5%.

**Conclusion:**

This article demonstrate that the linearity of the readout electronics affects the microdosimetric spectra with a difference in ${\bar{y}}_{F}$ values between the different linearization methods of up to 17.5%. The combined uncertainty was dominated by the uncertainty of stopping power on the edge.

## 1 INTRODUCTION

In modern radiotherapy, the use of high linear energy transfer (LET) particles has become more common, for example, in boron neutron capture therapy, proton therapy, or heavy ion therapy. Two benefits of the exploitation of ion beams are that the ratio of the sensitivity of oxygenated cells compared to hypoxic cells is drastically reduced and that the proximal dose of the tumor is lower as compared to low LET radiation. For low LET radiation, the variation in relative biological effectiveness (RBE) is small while for high LET the differences are not negligible. While there exists a correlation between RBE and LET, it has been shown that LET alone cannot predict the biological effect for a given endpoint.^1^ Experimental microdosimetry offers a method to distinguish components of different LET in a mixed radiation field, and microdosimetric quantities have been shown to correlate uniquely to RBE.^2^ Indeed, microdosimetry is of high interest for the radiation quality description of high LET radiation therapy beams.

The research community is facing a relevant challenge for applying microdosimetry in ion‐beam therapy, which is to provide a univocal interpretation of the physical characteristics of the irradiation. The objective is to provide an unambiguous representation of the radiation quality despite a significant difference between the used microdosimeters, in particular their shape and active volume material. Intense programs are underway at the MedAustron ion therapy center and elsewhere.^3^, ^4^, ^5^, ^6^, ^7^ The objective is to study the attributes of the spectra obtained with different microdosimeters under proton and carbon‐ion irradiation in an attempt to provide univocal and detector‐independent outcomes.

In the last decade, there has been interest in microdosimetric characterization of carbon‐ion beams with gas^8^ and solid‐state detectors.^5^, ^9^ In order to estimate microdosimetric quantities from those microdosimeters, a lineal energy calibration procedure of the electronic chain coupled to the detector has to be performed. Such calibration can be achieved either by an alpha source^10^ or by the so‐called edge technique.^3^, ^10^, ^11^ In the latter, a marker point is identified^12^ in the measured pulse‐height spectrum (PHS) and a specific lineal energy *y* is assigned to this marker point. To relate the counts in a specific channel of the multi‐channel analyzer (MCA) to the PHS value and thus to a lineal energy *y*, a linearization of the electronic chain must be performed along with the calibration in lineal energy. In some previous investigations, the calibration in terms of pulse amplitude is performed using only few points. Knowing the amplitude of the input pulse, a voltage divider is used so the pulse amplitude is reduced to a few values and a linear regression is then performed to correlate the channels to the respective pulse amplitude.^13^, ^14^

Whether this assumption about the electronics linearity holds true, especially for low voltage input signals, has not been studied yet as well as their uncertainties assessment.

Another relevant aspect of microdosimetry is that two different types of microdosimeters yield different spectra.^3^, ^15^ A conversion of the shape, material, and size of the sensitive volume (SV) can be performed to compare the microdosimetric spectra obtained under the same irradiation conditions by different microdosimeters.^16^, ^17^ This conversion allows to obtain the microdosimetric quantity for a tissue‐equivalent volume while the SV of the microdosimeter can be made of non‐tissue‐equivalent material or to compare two non‐tissue‐equivalent microdosimeters. The influence of different stopping power tables on the conversion between two solid‐state microdosimeters is also yet to be investigated.

This work describes a comparison of the responses of two different solid‐state microdosimeters, that is, diamond and silicon microdosimeters, irradiated under the same conditions and at the same depth in a carbon‐ion beam. The impact of different calibrations of the input‐voltage dependence of the multichannel scale and the use of different stopping power tables and their associated uncertainties are carefully investigated.

## 2 MATERIALS AND METHODS

### 2.1 Diamond and silicon microdosimeter structure

**FIGURE 1:**
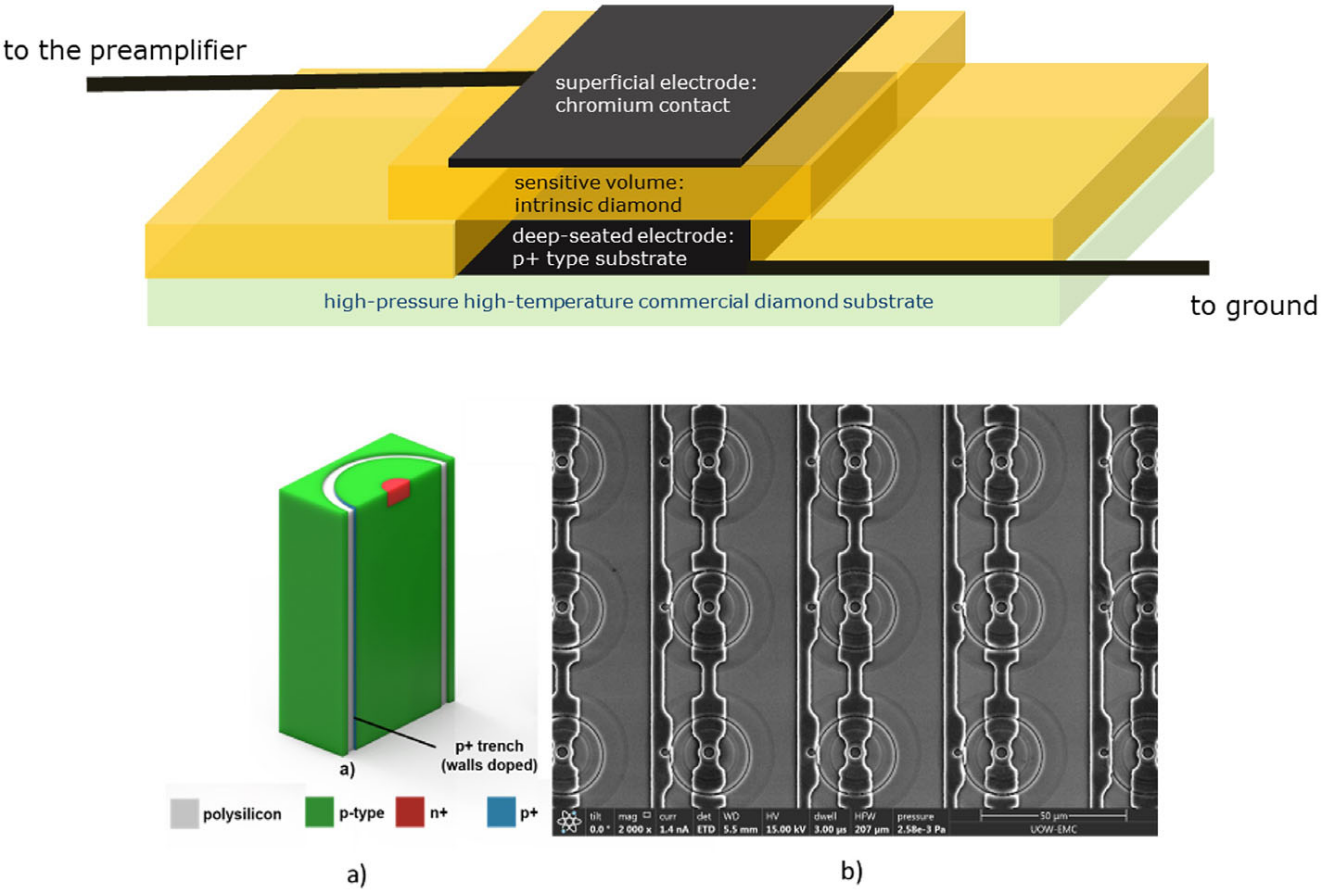
Top picture schematic representation of the diamond microdosimeter, bottom picture silicon microdosimeter, (a) simplified schematics illustrating sensitive volume geometry of a trenched planar structure and (b) scanning electron microscope image of the mushroom microdosimeter, adapted from B. James et al., “SOI Thin Microdosimeters for High LET Single‐Event Upset Studies in Fe, O, Xe, and Cocktail Ion Beam Fields,” in IEEE Transactions on Nuclear Science, vol. 67, no. 1, pp. 146‐153, Jan. 2020, https://doi.org/10.1109/TNS.2019.2939355

The two types of microdosimeters used in this work are slab microdosimeters with different geometries. The so‐called “3D mushroom” silicon microdosimeter^18^ was developed at the Center for Medical Radiation Physics (CMRP) of the University of Wollongong, Australia, and the diamond microdosimeter^19^ was developed at the industrial engineering department laboratories of the Tor Vergata University, Rome, Italy. A schematic representation of the two above mentioned microdosimeters is pictured in Figure 1.

The 3D mushroom microdosimeter used in this work consists of an array of 400 SVs. The SVs, having a diameter of 18 µm each, are fabricated on a high resistivity *p* type silicon on insulator (p‐SOI) active layer with a 10 µm thickness attached to a low resistivity supporting wafer with 2 µm silicon oxide between these two layers. Details on the device fabrication technology can be found in reference.^20^

The diamond microdosimeter has a multilayered structure obtained by a two‐step growing procedure through microwave plasma‐enhanced chemical vapor deposition (MWPECVD) technique. The diamond microdosimeter is a boron doped/intrinsic diamond/Cr Schottky diode. The detector is embedded in a metallic waterproof cylindrical housing filled by epoxy resin. The diamond SV of the microdosimeter used for this work is 200 µm × 200 µm × 2 µm. Details of the diamond microdosimeter design are reported elsewhere.^21^

### 2.2 Experimental setup

**FIGURE 2:**
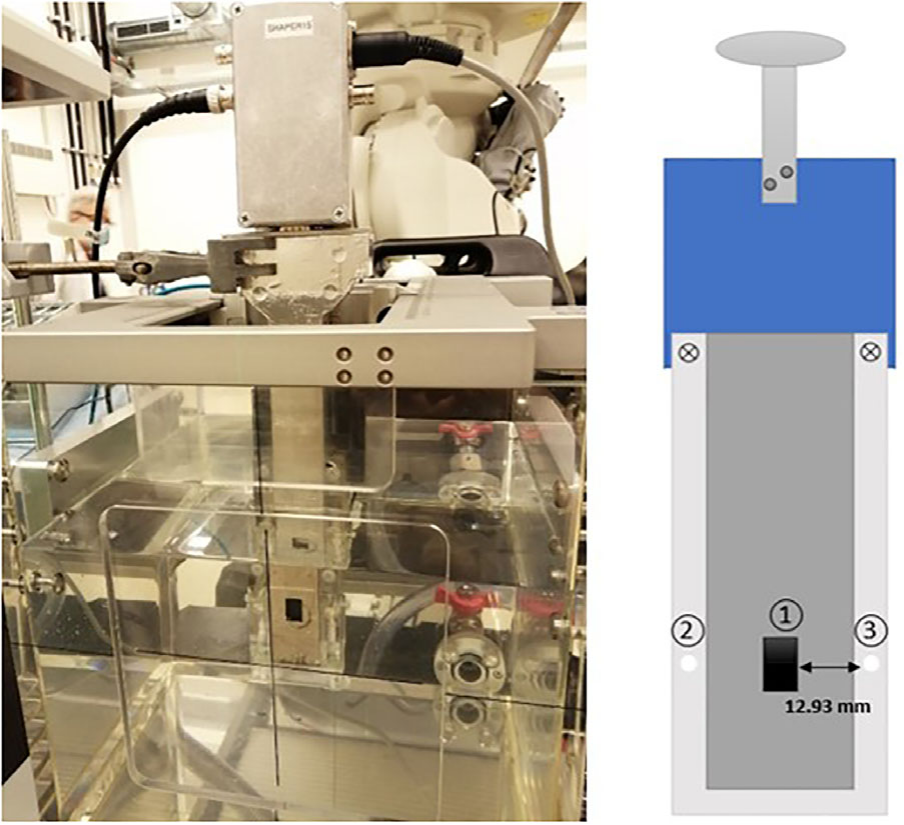
Left: the stationary water phantom PTW‐41023 with the detector holder positioned in water, right: schematic of the in‐house holder designed to align the three detectors at the same depth and to measure their response at the same time. The lateral distance between the silicon detector and the holes is 12.93 mm. (1) Silicon microdosimeter, (2) hole for microDiamond, (3) hole for diamond microdosimeter

The measurements were performed in a monoenergetic carbon‐ion beam with an energy of 284.7 MeV u^−1^. An in‐house developed sample holder for the stationary water phantom (of type PTW‐41023, PTW Freiburg, Germany) was designed to position both solid‐state microdosimeter types aligned at the same water depth, the silicon‐based microdosimeter in the center and diamond dosimeter (microDiamond, PTW‐60019, PTW Freiburg, Germany) as well as diamond microdosimeter from either side with a distance of 12.9 mm (see Figure 2). The PTW microDiamond is 1 µm thick with a radius of 1.1 mm. Before each acquisition of microdosimetric spectra, the diamond dosimeter was used to determine the depth dose profile and the position of the Bragg peak at the central axis. Then the microdosimeters were inserted into the holder. The diamond microdosimeter was aligned in depth to the silicon microdosimeter taking into account the water‐equivalent thickness (WET) values of the phantom and detector components in front of the sensitive volume.

The measurements were carried out at nine water depths along the pristine Bragg peak (14.9 cm). One position in the plateau at a depth in water of 10.9 cm and eight positions at different depths around the Bragg Peak, including the distal fall‐off region, were chosen. For several spectra, the measurements were repeated after four months to check the stability of the detector, and estimate the uncertainty associated with the reproducibility of the measurements.

### 2.3 Experimental approach

#### 2.3.1 Linearization

**FIGURE 3:**
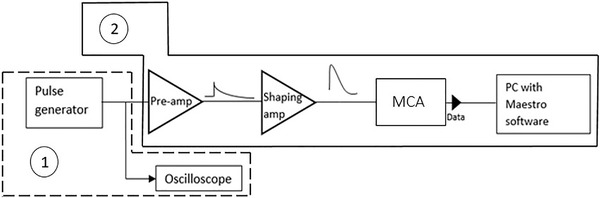
The schematic setup for the linearity check. The components indicated with (1) are used to measure the input test value provided by the pulse generator “TGF4242 AIM‐TTI” and the oscilloscope “PicoScope 4227.” The components indicated with (2) are used to detect the pulse in the corresponding channel in the MCA, starting from the pre‐amplifier “A250CF CoolFET Amptek” to the shaping amplifier “ORTEC Model 671” and finally to the multichannel analyzer “928 MCB.” The components indicated with (2) are used to detect the pulse in the corresponding channel in the MCA

Linearization is the process of establishing the correlation between the charge injected using a voltage pulse generator (mV) to the dedicated test capacitance of the preamplifier and the channels in the PHS. The scope of the linearization is to avoid any distortion in the lineal energy *y* spectrum due to the nonlinearity in the conversion of the signal from the microdosimeter to the pulse‐height histogram arising from the electronic components, predominantly in the lower channel part. Figure 3 illustrates the set‐up used to establish this relation. First, a pulse generator “TGF4242 AIM‐TTI” is connected to an oscilloscope “PicoScope 4227” to provide a measurement of the input test voltage, the input signal used for the linearization was a ramp with rising time of the order of few ns and decay time of 1 ms (frequency of 1 kHz). The second electronic set is the detection of the same signal from the pulse generator going through the test input of the pre‐amplifier “A250CF CoolFET Amptek” to amplify the signal without adding extra noise. The signal is processed in the shaping amplifier “ORTEC Model 671” and converted to a quasi‐Gaussian pulse. Finally, the signal is collected in the MCA “928 MCB,” transforming the analog output from the amplifier to a digital quantity. The uncertainty given by the manufacturer for each technical component was considered in the uncertainty budget and found to be negligible compared to other uncertainties.

**FIGURE 4:**
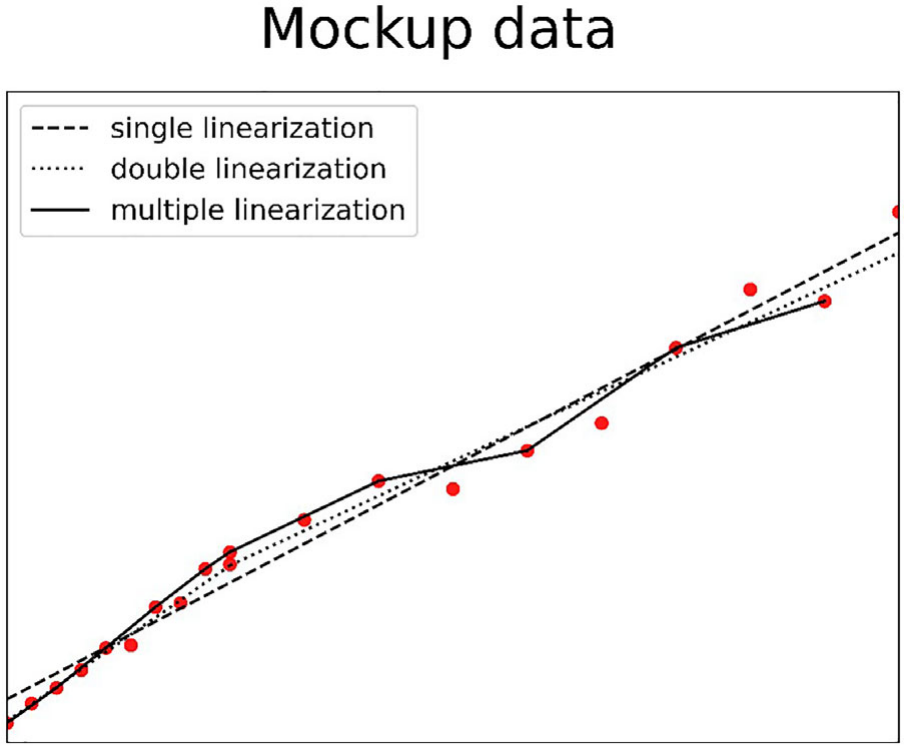
Illustration of the three linearization methods using mockup data exaggerating the fluctuations for visualization purposes: dashed single linearization, dotted double linearization, line multiple linearization

Multiple measurements were performed over a period of a few months to quantify the repeatability and reproducibility of the data. We started with dense pulse steps of 10 mV for the range between 10 and 500 mV, while above 500 mV the steps were reduced to 100 mV, so as to cover the entire range of amplitudes corresponding to the pulses from the detectors foreseen during the experiments. The same approach was followed starting from the highest to the lowest amplitude, resulting in negligible differences ruling out any hysteresis. Three linearization methods were investigated. Figure 4 illustrates those based on a mockup data set exaggerating the fluctuations for visualization purposes and does not represent the real experimental data used for linearization in this work for which it is difficult to see the features explained. The first method consists of a single linear regression Equation (1) to correlate the channels (*N*) with the corresponding pulse heights (*h*) in millivolts. The second consists of a double linear regression done by splitting the data into two intervals and applying linear fits connected by a hinge‐point for both data intervals, Equation (2) for the first interval, and Equation (2) for the second, then finding the best transition channel ($N_{t}$) from the first to the second linearization by minimizing the sum of squares of the residuals. The last method is a multiple linearization, the data were split into two groups of odd and even channels. The even channels were used for a linear fit according to Equation (3), while the odd channels were used to estimate the uncertainty of the fit by quantifying the residuals.

(1) h=a.N+b

(2) $$h=\left\{\begin{array}{cc} a_{1}N+b_{1}; & 0\leq N\leq N_{t}\\ a_{2}N_{2}+b_{1}+\left(a_{1}-a_{2}\right)N_{t}; & N>N_{t} \end{array}\right.$$

(3) $$\begin{array}{ccc} h & = & h_{2n+1}+\frac{N-N_{2n+1}}{N_{2n+3}-N_{2n+1}}\left(h_{2n+3}-h_{2n+1}\right);\\ N_{2n+1} & \leq & N\leq N_{2n+3} \end{array}$$

where *h* is the pulse height, *N* the channel number, $N_{t}$ the transition channel, and all other variables are fit parameters.

#### 2.3.2 Edge calibration

**FIGURE 5:**
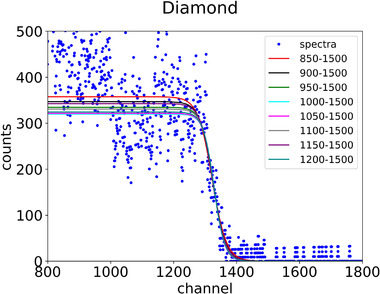
The Fermi‐like fit function with the various fitting ranges for the carbon edge using the diamond microdosimeter data

The position of the edge of the PHS is related to the maximum amount of energy that can be imparted by the primary ion in the SV of the detector. For a certain ion species, the edge value depends on the thickness of the microdosimeter, the material, and the direction of the beam in relation to the geometry of the microdosimeter. The calibration procedure in terms of energy imparted of the spectrum is based on identifying the edge value in the spectrum and associating the reference imparted energy to this value, that is, the maximum imparted energy in the continuous slowing down approximation (CSDA). The edge value can be assessed by fitting a sigmoid function, typically a Fermi‐like function, as shown in Equation (4), (see Figure 5 for the range between 1100 to 1500 channels), to the experimental data in the high gradient region corresponding to the maximum amount of energy imparted, that is, the edge region.

(4) $$h\cdot d\left(h\right)=\frac{A}{1+e^{B\left(h-C\right)}}$$

where h·d(h) represents the dose probability density of the pulse height *h* [mV] and A, B, and C are fit parameters.

The procedure was described by Conte et al.^12^ and is based on the possibility to recognize three marker points in the spectrum. The use of the marker point *h*_tc_, which is the intercept of the tangent through the inflection point with the horizontal axis (Equation 5), was shown to be the least affected by the choice of the fitting range and the counting statistics.^12^, ^22^ For this reason, the position *h*_tc_ was used in this study.

(5) $$h_{tc}=\frac{2}{B}+C$$

**FIGURE 6:**
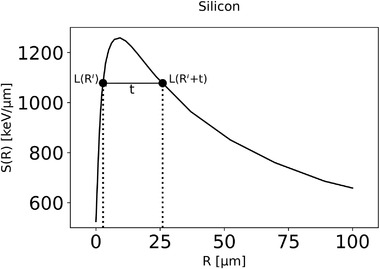
The unrestricted electronic stopping power curve as function of the range *R*, and an illustration of how to find the maximum value of the most probable energy loss corresponding with the range difference equal to the thickness (*t*) of the microdosimeter. (Note: *Rʹ* and *Rʹ* + *t* are the continuous slowing down approximation ranges in the medium made of material of the sensitive volume)

As already mentioned above, the reference edge energy, ε_max_, is commonly presumed to be the maximum energy that can be imparted in the CSDA.^3^ Therefore, ε_max_ is obtained as the product of *l*_max_ and *L*_max_, where *l*_max_ is the largest chord length and *L*_max_ is the maximum value obtained from the electronic stopping power lookup tables. This is true under the assumptions that the particle trajectories are straight, the energy loss straggling is negligible, there is no escape of delta rays, and the microdosimeter thickness is small enough such that the variation of LET of the particle during the traversal is negligible. The maximum value of lineal energy *y*_max_, is then obtained as follows^23^:

(6) $$y_{\max}=\frac{\varepsilon_{\max}}{\bar{l}}$$

where $\bar{l}$ is the mean chord length of the SV of the detector. However, in the case of a large SV, the LET is not constant during the transversal. In those cases, *y*_max_ is obtained considering the variation of LET when traversing the microdosimeter. Typical curves of electronic stopping powers as function of the particle range can be obtained from look‐up tables.^24^, ^25^ For carbon ions and the relevant materials (water, graphite, amorphous carbon, silicon), the electronic stopping power increases, then reaches a maximum at the energy of approximately 5 MeV and then monotonically decreases. As per ICRU report 85^26^ definition, the unrestricted electronic stopping power is equal to the unrestricted LET (indicated in the equation with the letter L). In Figure 6 we emphasize the role of the two quantities using S for representing the electronic stopping‐power, and L for the microdosimetric edge computation

Equation (6) can be re‐written as follows:

(7) $$y_{\max}=\frac{l_{\max}}{\bar{l}}\cdot L_{\max}$$

For very thin SVs (where the product between the thickness *t* and the material density *ρ* is less than 1 µm g cm^−3^), *L̅*_max_ can be approximated using the maximum value of the stopping power, *L*_max_, which corresponds to the value of the peak in Figure 6. On the other hand, for very large SVs (where *t*·*ρ* ≥ 20 µm g cm^−3^) like in the 3D SOI microdosimeter, *L̅*_max_ can be calculated by dividing the energy corresponding in the lookup table to a range equal to the thickness of the detector *t* by *t* itself.

For intermediate SV thicknesses, typical of various solid‐state microdosimeters, a more appropriate method must be adopted. Assuming that the SVs are slabs, we follow a similar methodology as reported by Chiriotti^27^ but instead of finding $\Delta E_{\max}$ numerically, we formally identify it as follows.

Let *R* be the free parameter which indicates the range when the particle exits the SV. All ion trajectories are assumed to be parallel, and normally incident to the slab SV. In a slab microdosimeter, this comprises all the ranges of a particle crossing the sensitive volume. The value of LET, averaged over the thickness of the SV, can be expressed as a function of *R*:

(8) $$\begin{array}{ccc} \bar{L}\left(R,t\right) & = & \frac{1}{t}\int_{R}^{R+t}L\left(x\right)dx=\frac{1}{t}\int_{R}^{R+t}\frac{dE}{dx}dx\\ & = & \frac{E\left(R+t\right)-E\left(R\right)}{t} \end{array}$$

where the identity between LET and unrestricted electronic stopping power is expressed, within the integral, by *x*, *L(x)* = *dE*/*dx*, and the energy lost in elastic nuclear reactions and in Bremsstrahlung is assumed to be negligible. The next step is to identify the maximum value of *L̅(R, t)* Equation (8) and its corresponding range $R{}^{\prime }$. For a specific thickness t and as long as *L̅(R, t)* is expressed by a concave downward function of R, the maximum value *L̄*_max_ is obtained when its derivative of $\bar{L}\left(R,t\right)$ is zero:

(9) $$\begin{array}{ccc} \frac{d\bar{L}\left(R,t\right)}{dR} & = & \frac{1}{t}\frac{d}{dR}\int_{R}^{R+t}L\left(R\right)dx\\ & = & \frac{1}{t}\left[L\left(R+t\right)-L\left(R\right)\right]=0 \end{array}$$

Therefore, the maximum of *L̅(R, t)* occurs at the value of the range, *R'*, for which the segment *t* in Figure 6 is parallel to the axis *R*, or in other words, the LET at entrance and exit are equal.

Substituting *L*_max_ with $\bar{L}\left(R,t\right)={\bar{L}}_{\max}$ and taking into account that, for parallel trajectories incident normally to the slab detector, *t*, $\bar{l}$, and *l*_max_ coincide, Equation (7) using Equations (8) and (9) can be re‐written as:

(10) $$y_{\max}={\bar{L}}_{\max}=\frac{E\left(R^{\prime }+t\right)-E\left(R^{\prime }\right)}{t}$$

The use of Equation (10) reduces the uncertainty in the determination of *L̅*_max_ when using the thin or thick detector approach described above, which, in the interval 1 µm g cm^−3^ ≤ *t·ρ* ≤ 20 µm g cm^−3^, can be estimated from the stopping power table to be up to 7%.

The calibration factor *K*_cal_ is calculated by dividing the maximum value of lineal energy Equation (10) by the value of the marker point found using Equation (5)

(11) $$K_{\mathrm{cal}}=\frac{y_{\max}}{h_{tc}}$$

This calibration factor is uniformly applied to the linearized channels. At this point the horizontal axis in the microdosimetric spectra representation corresponds to the lineal energy *y* [keV µm^−1^].

#### 2.3.3 Microdosimetric values

Building on the definition of the real ${\bar{y}}_{D}$ given by Lindborg and Waker,^28^ in this paper, the dose mean lineal energy ${\bar{y}}_{D}$ is calculated above the cutoff due to the electromagnetic noise and since we cannot determine the dose distribution experimentally below the cutoff, we can only normalize the distribution *d*(*y*) above the cutoff and calculate the ${\bar{y}}_{D,a}$ (where “*a*” is the cutoff channel) as follows:

(12) $${\bar{y}}_{D,a}=\int_{a}^{y_{\max}}yd\left(y\right)dy$$

In the same way, ${\bar{y}}_{F,a}$ is calculated above the cutoff channel, as follows:

(13) $${\bar{y}}_{F,a}=\int_{a}^{y_{\max}}yf\left(y\right)dy$$

### 2.4 Spectrum transformation between different microdosimeter materials

Four different methods were studied for the material transformation to convert the microdosimetric spectra obtained by silicon detector into spectra we would expect to obtain from a diamond detector, and vice versa. The first three use a single value to adjust the position of the edge and rescale the data. The fourth method converts the lineal energy from one material to another for each bin using tabulated stopping power data. Since no database reports stopping power values for diamond, the values for amorphous carbon from ICRU look‐up tables and graphite from SRIM tables are used instead.

Method 1, referred in this work as the “edge method,” consists of simply using the maximum value of lineal energy for the diamond microdosimeter instead of the maximum value for the silicon microdosimeter.^3^ In detail this means that the lineal energy of the edge value of a 2 µm thick diamond slab (ICRU 730.52 keV µm^−1^, SRIM 677.32 keV µm^−1^) is used instead of the original edge value corresponding to a 10 µm thick silicon slab (511.26 keV µm^−1^).

Method 2, referred in this work as the “density method,” is based on the material densities. We assume that the product of the material's thickness and density is equal for the two investigated materials^2^ forcing that the two detectors have the same atomic composition. In this case, the diamond microdosimeter's thickness *t*_d_ is given by

(14) $$t_{d}=\frac{t_{s}\cdot \rho_{s}}{\rho_{d}}.$$

where *t*_s_ is the silicon microdosimeter's thickness (10 µm), ρ_s_ its density (2.32 g cm^−3^), and ρ_d_ is the diamond microdosimeter's density (3.52 g cm^−3^), thus *t*_d_ = 6.6 µm.

Method 3, referred in this work as the “maximum‐deposition energy method,” consists of considering that the maximum energy deposition is the same in both SVs of the silicon and diamond microdosimeters.^29^ We take the edge energy value defined by the silicon SV thickness, then change silicon to diamond and adjust the thickness of the diamond SV until the maximum deposited energy by carbon ion is the same as maximum energy deposited in silicon. The maximum energy imparted by carbon ions in a 10 µm thick silicon slab microdosimeter, assuming the CSDA, are 11794.3 keV (10628.2 keV) for ICRU (SRIM) look‐up tables. The same maximum energy is imparted to a diamond microdosimeter with a thickness of 4.96 µm (4.65 µm).

Method 2 assume that the ratio of the electronic stopping power of the two materials is independent of the particle type and energy,^29^ while methods 1 and 3 assume that the ratio of the electronic stopping powers of the two materials is dependent of the energy and particle type^2^, ^3^ only at the edge region. These assumptions are not completely fulfilled when silicon and diamond are compared, rather the ratio of the electronic stopping power is dependent of the energy and particle type for all lineal energy values.

Method 4, referred in this work as the “bin‐by‐bin transformation method,” was explained by Magrin.^17^ This method takes into consideration the dependence of the ratio of the electronic stopping powers of the two materials with the particle energy and it is based on the assumption that, for a particular bin, the lineal energy value measured with silicon microdosimeter can be substituted with a closest LET(Si) value; the energy of ions corresponding to this LET(Si) is then determined from the look up table of one of the databases. For each obtained ion energy, the LET(diamond) is determined by interpolation from the database of the diamond LETs and the corresponding ion energies. The ratio LET(Si)/LET(diamond) is then used for the conversion of the silicon microdosimetry spectrum to diamond for a particular bin. This method requires that the energy‐loss straggling and the delta‐ray escape from the SV are small. These conditions are not satisfied at the highest beam energies corresponding to measurements at the plateau of the Bragg curve. However, if the two microdosimeters have SVs with similar thickness and transversal sizes, the energy‐loss straggling and the delta‐ray escape are also compatible and the pristine and the converted spectra are expected to be similar.

### 2.5 Uncertainty estimation

The uncertainty was estimated for all linearization methods to highlight their influence on the spectra and their propagation to the uncertainties of the value of ${\bar{y}}_{F}$ and ${\bar{y}}_{D}$. All reported uncertainties are expressed at the level of one standard deviation. For the single linearization method, the uncertainty was calculated by obtaining uncertainties on the slope and intercept (channel number) resulting from the least‐squares linear regression. The double linearization method's uncertainty was estimated separately for both linear regressions (below/above the transition channel). The multiple linearization uncertainty was estimated by quantifying the residuals between the data points not used in the fit (see Section 2.3.1).

The uncertainties of the marker point corresponding to the carbon edge for both microdosimeters were estimated by using the method described by Parisi et al.^22^ for the experimental data. Different fitting ranges were applied for the Fermi‐like fit starting from 850, 900, 950, 1000, 1050, 1100, 1150, or 1200 to 1500 mV for the diamond spectrum. The silicon spectrum was fitted from 1500, 1550, 1600, 1650, 1700, 1750, 1800, 1850, 1900, 1950, or 2000 to 2400 mV.

The uncertainty on the stopping power tables of different materials may have some level of correlations if the stopping power data in ICRU report 49^30^ comes from correlated sources. So, combining uncertainties of two stopping power tables directly could be an overestimation. For the diamond microdosimeter, the edge uncertainty contributes to the uncertainty of ${\bar{y}}_{F}$ and ${\bar{y}}_{D}$ values, but when the silicon microdosimeter data are converted to diamond‐equivalent data, the uncertainties were estimated without taking the correlation into account.

The uncertainty on the SV thickness of the microdosimeters was also taken into account. The propagation of this uncertainty, to ${\bar{y}}_{F}$ and ${\bar{y}}_{D}$ values, is a tolerance level, and was considered as 100% confidence interval limits of a rectangular distribution. The standard deviation was calculated by dividing the tolerance by the square root of 3.

## 3 RESULTS AND DISCUSSION

**FIGURE 7:**
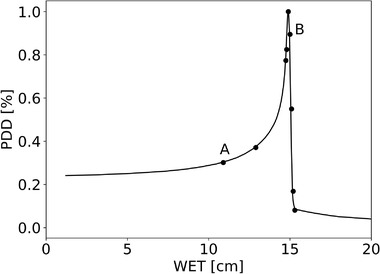
Depth dose profile of the 284.7 MeV u^−1^ carbon‐ion beam, and the nine measurement positions where microdosimetric spectra were obtained with the diamond and silicon microdosimeter

**FIGURE 8:**
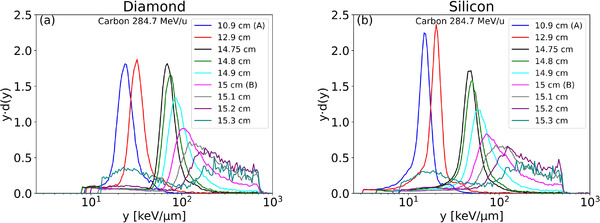
Microdosimetric spectra measured at different positions along the Bragg peak curve in a carbon‐ion beam using the double linearization; (a) diamond microdosimeter experimental data, (b) silicon microdosimeter experimental data

The depth dose profile of the 284.7 MeV u^−1^ carbon‐ion beam, and the nine positions measured by the microDiamond are shown in Figure 7 where A is the first position in the plateau and B is R_90_ corresponding with the 90% depth dose level in the distal part of the Bragg peak curve. All the values of ${\bar{y}}_{F}$ and ${\bar{y}}_{D}$ and their uncertainty contributions are shown in Tables 1, 2, 3. The details, some graphically illustrated, are described and discussed below. According to ICRU 73^31^ and SRIM 2013^25^ tables and considering the silicon detector thickness of 10 µm, the lineal energy of carbon ions according to Equation (12) is 511.26 and 456.27 keV µm^−1^, respectively. In diamond, for a range of 2 µm, the carbon edge is 730.52 and 677.32 keV µm^−1^, respectively. The position of the edge for the silicon detector spectra was found to be 844.46 mV and 83.98 mV for the diamond detector. Figure 8 presents the calibrated microdosimetric spectra obtained by the diamond microdosimeter (a) and the silicon microdosimeter (b) for different water depths before the detector material conversion, using the double linearization. In the horizontal axis, the lineal energy is represented by scaling the values to the density of 1 g cm^−3^. The type A uncertainty of measurements taken again after four months was found to be negligible on all relevant parameters such as the channels, the dose maximum, the edge, the mean values.

**TABLE 1:** ${\bar{y}}_{F}$ and ${\bar{y}}_{D}$ values in keV µm^−1^ and their linearization uncertainty (related to original microdosimetric spectra in diamond and silicon)

**Diamond microdosimeter**	**Single linearization**		**Double linearization**		**Multiple linearization**	
**Depth**	10.9 cm	15.0 cm	10.9 cm	15.0 cm	10.9 cm	15.0 cm
${\bar{y}}_{F}$	25.27 ± 1.19	84.65 ± 1.13	23.31 ± 0.45	82.75 ± 0.41	24.39 ± 4.73	84.06 ± 4.73
${\bar{y}}_{D}$	29.04 ± 1.01	166.18 ± 0.09	27.40 ± 0.37	166.32 ± 0.17	28.33 ± 3.93	167.15 ± 0.04

**Silicon microdosimeter**	**Single linearization**		**Double linearization**		**Multiple linearization**	
**Depth**	10.9 cm	15.0 cm	10.9 cm	15.0 cm	10.9 cm	15.0 cm
${\bar{y}}_{F}$	15.28 ± 0.35	68.53 ± 0.30	13.01 ± 0.05	63.23 ± 0.05	13.16 ± 0.28	67.25 ± 0.28
${\bar{y}}_{D}$	17.50 ± 0.29	119.08 ± 0.03	15.70 ± 0.04	119.31 ± 0.04	15.80 ± 0.22	120.13 ± 0.06

**TABLE 2:** ${\bar{y}}_{F}$ and ${\bar{y}}_{D}$ values in keV µm^−1^ measured with the diamond microdosimeter and with the silicon microdosimeter while converted to diamond with methods 1–4 in the plateau region position A (depth 10.9 cm) and their uncertainty propagation (using the double linearization) for the marker point, the stopping powers, and the thickness

**Quantity [keV µm^−1^]**	**Diamond**	**Method 1**	**Method 2**	**Method 3**	**Method 4**
${\bar{y}}_{F}$ value	23.31	20.25	18.36	19.01	16.08
u marker point	0.03	0.01	0.01	0.01	0.01
u stopping power	1.17	1.01	0.95	0.95	1.14
u thickness	0.06	0.06	0.08	0.08	0.04
${\bar{y}}_{D}$ value	27.40	23.09	20.78	21.57	18.24
u marker point	0.04	0.02	0.01	0.02	0.01
u stopping power	1.37	1.15	1.04	1.08	1.29
u thickness	0.08	0.06	0.10	0.08	0.05

**TABLE 3:** ${\bar{y}}_{F}$ and ${\bar{y}}_{D}$ values in keV µm^−1^ measured with the diamond microdosimeter and with the silicon microdosimeter while converted to diamond with methods 1–4 in the R_90_ (depth 15.0 cm) and their uncertainty propagation (using the double linearization) for the marker point, the stopping powers, and the thickness

**Quantity [keV µm^−1^]**	**Diamond**	**Method 1**	**Method 2**	**Method 3**	**Method 4**
${\bar{y}}_{F}$ value	92.12	101.33	93.71	96.00	86.85
u marker point	0.13	0.07	0.07	0.07	0.07
u stopping power	4.61	5.07	4.69	4.8	6.14
u thickness	0.26	0.29	0.44	0.37	0.24
${\bar{y}}_{D}$ value	168.47	172.62	154.89	160.83	146.47
u marker point	0.23	0.12	0.11	0.12	0.12
u stopping power	8.43	8.63	7.74	8.04	10.36
u thickness	0.48	0.49	0.73	0.62	0.42

**FIGURE 9:**
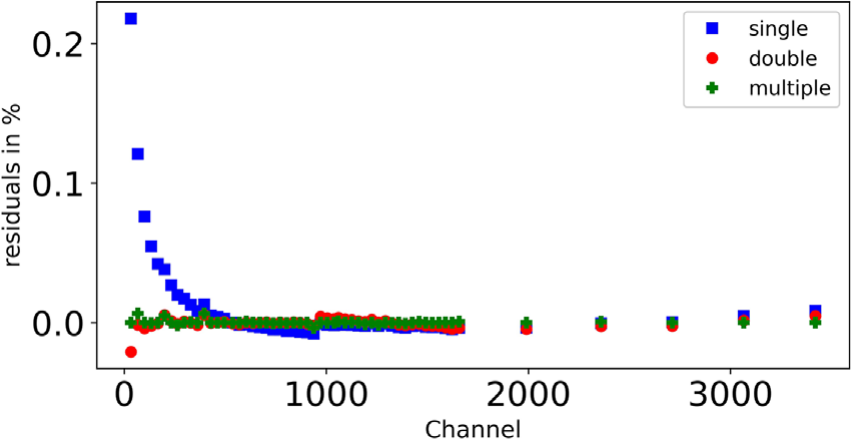
The residuals in percentage of the different linearization methods plotted as function of the channels for the silicon microdosimeter electronics

Figure 9 illustrates the residuals in percentage of the different linearization methods (single, double, multiple). The transition channels for the double linearization between the first and second intervals (as explained in Section 2.3.1), were 871 and 971 out of 4096 channels for the silicon and diamond microdosimetry respectively. It is a general practice to use a single‐point check of linearity using a high precision pulse generator and divider (as explained in Section 1) despite the high residuals for the single linearization in particular in the first channels. This procedure may lead to large relative discrepancies at the lowest channels and in the intercept. For the double and multiple linearization, the difference is only seen in the first two points. Above channel 500, the three methods agree within 0.014%.

**FIGURE 10:**
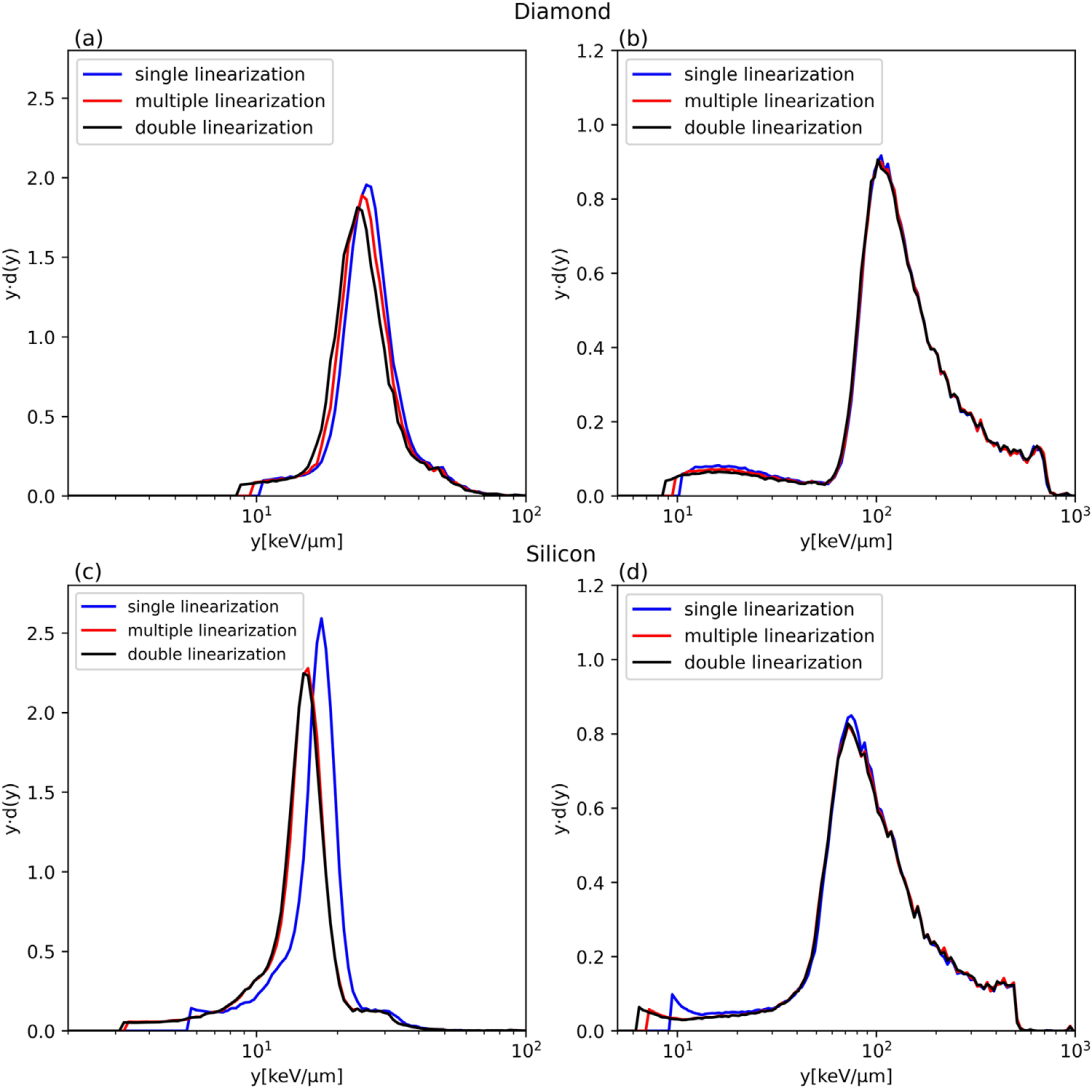
Microdosimetric spectra at two different depths 10.9 cm (left) and 15.0 cm (right) obtained with the diamond (up) and silicon microdosimeters (down) with the three different linearization methods

Figure 10 shows the lineal energy spectra resulting from the three linearization methods for diamond and silicon microdosimeters at two water depths, 10.9 and 15.0 cm. For the diamond microdosimeter in the plateau position A (10.9 cm), the microdosimetric spectra differ for the three methods (Figure 10a). As a general indication, the peak is moved by 3.9% between the single and the double linearization, and by 8.0% between the multiple and double linearization. At the position B R_90_ (15.0 cm) (Figure 10b), the difference is only visible for the lower lineal energy range. For the silicon microdosimeter, the spectrum obtained in the plateau position with using the single linearization is shifted to higher y‐values (15.28 keV µm^−1^, Table 1) in the lower lineal energy interval compared to the spectra obtained with the other methods (Figure 10c). As a general indication, the peak is moved by 16.6% between the single and the double linearization, and by 3.9% between the multiple and double linearization. For the R_90_ position B the variation of the microdosimetric spectra is like the diamond microdosimeter for lineal energies about 10 keV µm^−1^ (Figure 10, d). This result is consistent with what we have already seen in Figure 9, that is., that the residuals for the single linearization are higher in the first channels (thus first mV), and for the double and multiple linearization, the residuals are smaller. In particular, the electronic linearization procedure affects the spectrum position in the low lineal energy values for both microdosimeters.

### 3.1 Spectrum transformation between different microdosimeter materials

**FIGURE 11:**
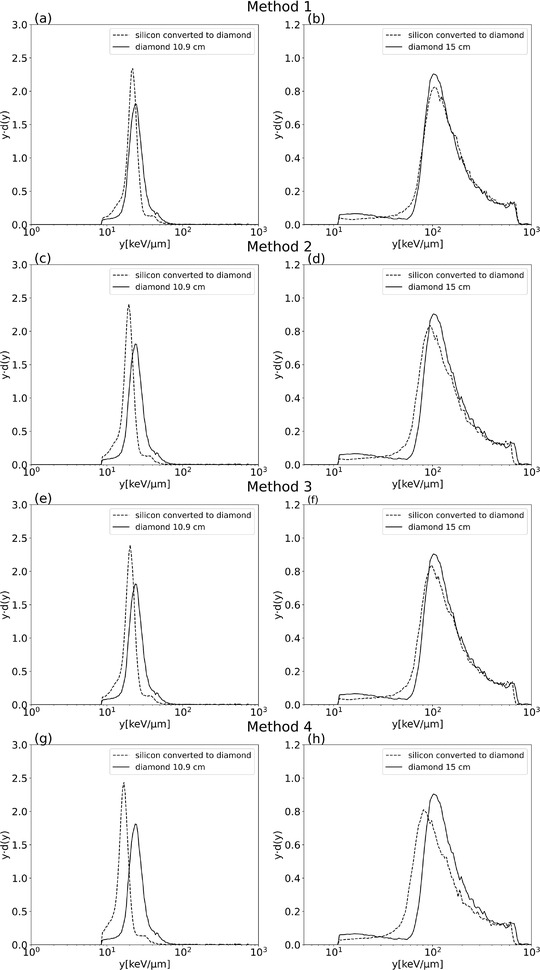
*yd*(*y*) distributions obtained with the diamond detector compared to silicon spectra converted to diamond using different conversion methods. (a) and (b) method 1 in the plateau (position A) at a depth of 10.9 cm and in R_90_ (position B) at a depth of 15.0 cm respectively, (c) and (d) method 2, (e) and (f) method 3, (g) and (h) method 4 for the same two positions

Figure 11 represents the spectra obtained with the different methods used to compare the microdosimetric spectra collected with the diamond microdosimeter with those obtained with the silicon microdosimeter converted to diamond one. Only the double linearization method and ICRU stopping power look up tables were used to calibrate the spectra. However, we have done the same exercise with the single linearization and with the SRIM stopping powers database resulting in very similar observations. The microdosimetric spectra were cut at the same lineal energy value to facilitate the comparison of the spectra and the values of ${\bar{y}}_{F,a}$ and ${\bar{y}}_{D,a},$ as explained in Section 2.3.3, represent the value of ${\bar{y}}_{F}$ and ${\bar{y}}_{D}$ above the cutoff channels (*a* = 8.76 and 11.46 keV µm^−1^ at the positions A and B). These values are reported in Tables 2 and 3 for diamond and silicon converted to diamond following the four methods. We recognize some level of pile‐up in the silicon spectra in the plateau as the second bump is around approximately double the first lineal energy peak due to its larger area (approximately 0.1 mm^2^) than diamond one, (approximately 0.04 mm^2^). While there are methods to mitigate the pile‐up, it is not a topic that we will address in this paper.

Differences between the two spectra are observed both in the plateau region and in proximity of the R_90_ for all the methods. In particular, the converted spectra are higher and narrower than those experimentally obtained with the diamond detector at 10.9 cm for all used conversion methods. This is due to the fact that the SV of the silicon microdosimeter is thicker than the diamond one and the number of primary collisions is higher. Under those conditions, the energy‐loss straggling of the silicon microdosimeter is smaller, in relative terms, than in the case of the diamond microdosimeter.

If we shift the edge (‘‘recalibrate’’) so that both spectra coincide (method one), we get a reasonable agreement (see Figure 11a,b); but the weakness of this method is that it does not take into consideration the thickness of the silicon microdosimeter. In the lower channels, we have a slight difference in the position of the spectra. The shape of the microdosimetric spectra for both microdosimeters at the R_90_ position (15.0 cm) looks quite similar, but looking at the mean values at this point (Tables 2 and 3), we see that the difference is mainly in ${\bar{y}}_{F}$ rather than in ${\bar{y}}_{D}$. The ${\bar{y}}_{F}$ value is more sensitive to the variation of the lowest values of the distributions while ${\bar{y}}_{D}$ is more sensible to the variation at the highest values. By deriving microdosimetric spectra obtained with the silicon microdosimeter while renormalized to match the edge of the spectra obtained with the diamond microdosimeter, spectral differences are minimized in the region of the highest lineal energy values.

Although the second and third methods (see Figures 11c and 11e, respectively) show similar results in the plateau, we see that these methods differ in the R_90_ position (see Figures 11d and 11f, respectively. In method three, the silicon spectra move more toward the diamond spectra, which is more evident in the value of ${\bar{y}}_{F}$ and ${\bar{y}}_{D}$, that is, their values are closer to the ones of the diamond microdosimeter with method 3 than with method 2.

When using method 4, the difference in the spectra is more evident (see Figure 11g,h). As discussed above, this method relies on the correspondence of the lineal energy with the LET. This condition is not fulfilled at the high ion‐beam energies due to the delta ray escape. The escape of delta rays also differs for the two microdosimeters at high ion‐beam energies due to the relevant differences in SVs shapes. The spectra from the detector with narrower transversal size underrepresent the high‐energy carbon‐ion losses and the peaks are moved toward lower lineal energies. Approaching the position of the Bragg peak, the effect of the delta‐ray escape decreases and so does the difference between the spectra, while still significant.

The use of a single conversion value (such as in methods 1, 2, and 3) does not consider the difference of the ratio of the stopping power between the materials within the energy interval. The stopping‐power values refer to the edge and therefore to very low energies. At those carbon‐ion energies, the stopping‐power ratio of diamond to silicon is approximately 25% higher than the value at the highest carbon‐ion energies. Since it is maintained constant for all energies, the effect is to move the spectra obtained at the plateau to higher lineal energy values.

Furthermore, in method 1, the spectra obtained with the silicon microdosimeter are moved further to the right side to align the edge with the edge obtained from the diamond microdosimeter.

These observations partially explain how, unexpectedly, the most approximate method 1, is the one for which the conversion appears to be in agreement visually in terms of spectra and parametrically also for the values ${\bar{y}}_{F}$ and ${\bar{y}}_{D}$. The better overlap is the consequence of the compensation of three separate distortions: the larger escape of delta rays in the silicon microdosimeter, the use of a constant ratio of silicon's and diamond's stopping powers for all energies, and the effect of the larger thickness of the silicon detector in the assessment of the carbon‐ion edge. The first distortion partially compensates the other two.

The presented analysis is complicated by the fact that the shape of the SVs of the silicon (a cylinder of 18 µm diameter and 10 µm thick) and diamond (slab with a square base 200 µm × 200 µm and 2 µm thick) microdosimeters are substantially different. The delta ray escape is related to the geometry of the SV and the number of primary collisions correlated to the thickness of the SV. Both effects have an influence on the shape of the microdosimetric spectra. While it is at present unclear if the good agreement with method 1 is the result of a coincidental compensation for this particular combination of detectors or if there is a more generic systematic mechanism behind it, it is of interest to investigate if this could be employed as a general method to design a microdosimeter from a different material as the site for which the microdosimetric spectrum has to be known. Monte Carlo simulation would be the preferential tool to investigate this in a systematic way.

### 3.2 Uncertainty budget

**FIGURE 12:**
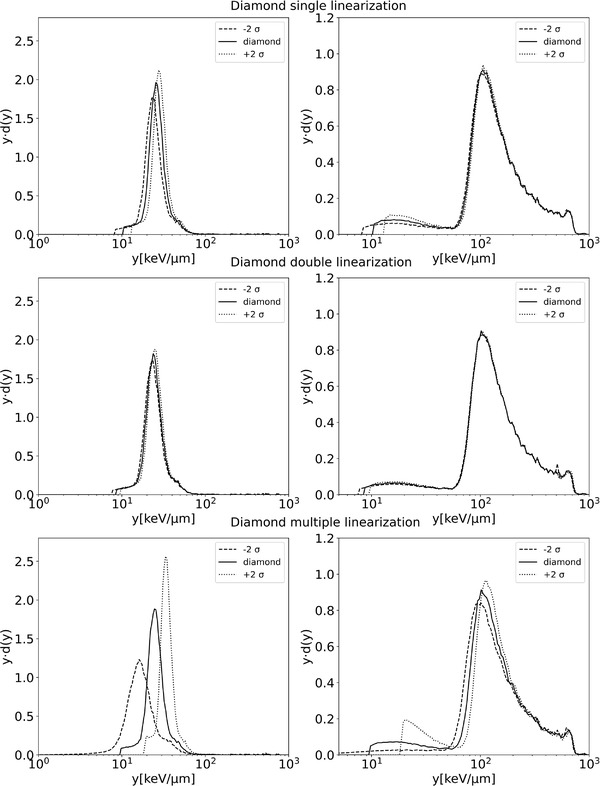
Diamond microdosimeter uncertainty envelopes with a 95% confidence interval of the *yd*(*y*) distributions with the different linearization methods, plateau left columns, and R_90_ right columns

**FIGURE 13:**
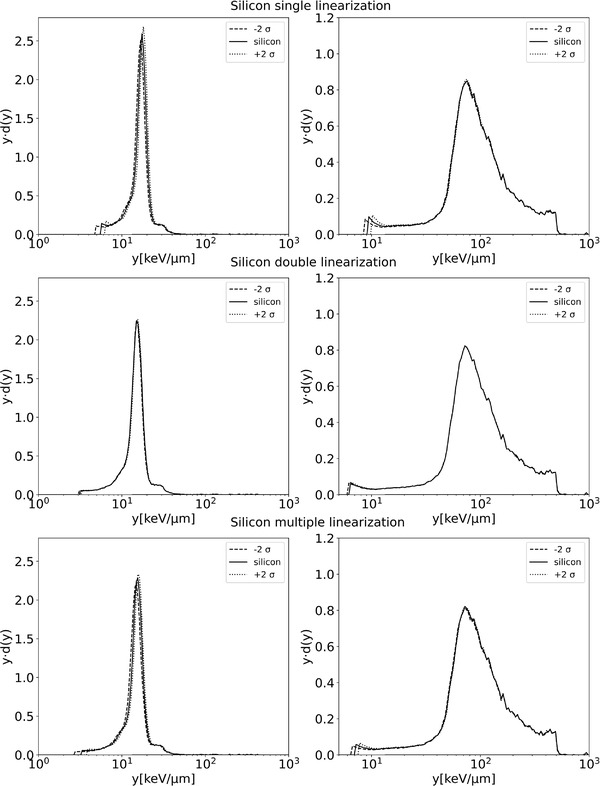
Silicon microdosimeter uncertainty envelopes with a 95% confidence interval of the *yd*(*y*) distributions with the different linearization methods, plateau left columns, and R_90_ right columns

Dose distributions of lineal energy obtained with the three linearization methods are reported in Figure 12 for the diamond microdosimeter and Figure 13 for the silicon microdosimeter. The uncertainty of the linearization is propagated to the shape of the microdosimetric spectra to emphasize the effect of the differences between the linearizations; spectra obtained for ±2 σ of the regression parameters are shown, which envelope a 95% confidence interval. There is a clear difference in the behavior of the two microdosimeters. The diamond microdosimeter uncertainty is more influenced by the different linearizations. The double linearization results in the smallest uncertainty on ${\bar{y}}_{F}$ and ${\bar{y}}_{D}$ in the plateau and the R_90_ (2% and 0.5%, respectively). The uncertainty for the single linearization is more significant in the plateau than in the R_90_. The multiple linearization has the highest uncertainty. The multiple linearization is not suitable for the diamond microdosimeter due to the noise in the data since the diamond microdosimeter do not have an electronic read out module close to the detector (but almost 1 m of cable to preamplifier). This inevitably increases the noise that will be directly propagated to the uncertainty, while for the linear and double linearization, the noise is smoothed out.

For the silicon microdosimeter, all three methods have smaller uncertainties as compared to the diamond microdosimeter. The multiple linearization has a lower uncertainty with the silicon microdosimeter than with the diamond microdosimeter, due to the low noise level, but also for the silicon, the double linearization has the lowest uncertainty of the three linearization methods.

The uncertainty contributions on ${\bar{y}}_{F}$ and ${\bar{y}}_{D}$, resulting from propagating the linearization uncertainty, are given in Table 1 for the diamond and silicon microdosimeters. Table 1 shows that first, as discussed in Section 2.3.1, the ${\bar{y}}_{F}$ and ${\bar{y}}_{D}$ values are very sensitive to the small differences resulting from the different linearization methods. Second, the uncertainties are always higher in the plateau, as shown in Figure 12; for example, for the multiple linearization in the diamond microdosimeter, the uncertainty of ${\bar{y}}_{D}$ value around the R_90_ is minimal. The uncertainties of ${\bar{y}}_{F}$ do not depend on depth while those for ${\bar{y}}_{D}$ do depend strongly on depth. The difference in ${\bar{y}}_{F}$ between the single and double linearization for the diamond microdosimeter is 8.4% and 4.6% between the multiple and double linearization. For the silicon microdosimeter, the difference is 17.5% between the single and double linearization, and 1.2% between the multiple and the double linearization.

Figure 5 shows the different fitting ranges applied for the Fermi‐like fit to find the marker point, even though the lower side of the sigmoid is different, the marker point is almost the same. The uncertainty of the marker point $h_{tc}$ was found by Parisi et al.^22^ to be 3.3% for their collimated proton beam. In this paper, for the carbon beam, the value of $h_{tc}$ with its standard uncertainty interval after fitting the spectra over different ranges for the diamond detector is 83.98 ± 0.12 mV (0.14%) while for the silicon detector the corresponding value is 844.5 ± 0.6 mV (0.07%), hence the relative uncertainty is lower. First, the carbon‐ion edge has a smaller uncertainty than the proton edge because it is sharper due to the significantly lower energy loss straggling. Second, the silicon microdosimeter uncertainty is lower than the diamond because the number of electron collision of the primary particles is significantly higher in a thicker microdosimeter.

The uncertainties of the electronic stopping powers given in ICRU Report 90,^32^ are used. The energy corresponding to the maximum energy loss in the CSDA approximation is between 4 and 5 MeV. ICRU report 90 specifies that for the region of 10–1 MeV the relative standard uncertainty is between 5% and 15%. Since we are closer to 10 MeV than to 1 MeV we have estimated the uncertainty to be 5%.

The uncertainty on the thickness of the silicon microdosimeter is 5% and for a very thin diamond active film the uncertainty is approximately 10%.

Tables 2 and 3 display the ${\bar{y}}_{F}$ and ${\bar{y}}_{D}$ values and their uncertainties resulting from the marker point, the stopping power databases and the SV thickness measured in the plateau region and the R_90_ respectively, using the double linearization. The relative uncertainties on ${\bar{y}}_{F}$ and ${\bar{y}}_{D}$ resulting from the uncertainties of the marker point in the plateau are 0.13% and 0.15%, respectively (see Table 2), for the diamond microdosimeter spectra. For the conversion methods, the ${\bar{y}}_{F}$ uncertainty is 0.05% in the first three methods, while in method 4 the uncertainty goes up to 0.06%. The ${\bar{y}}_{D}$ uncertainty fluctuates between 0.05% and 0.09% in the conversion methods. In the Bragg peak (see Table 3), the marker point uncertainty propagates to similar values with 0.14% and 0.07% for the diamond and all the first three methods respectively, and 0.08% for method 4. The propagated uncertainty of the stopping power is about 5% for the diamond microdosimeter and the first three methods of detector material conversion whilst for method 4 it is significantly higher (up to 7.1%). The propagation of the thickness uncertainty varies among the different methods between 0.25% and 0.49%.

The total uncertainty resulting from the linearization, the marker point, the stopping power data bases as well as the thickness of the SV was also estimated. The uncertainty on the ${\bar{y}}_{F}$ and ${\bar{y}}_{D}$ is about 5% for the diamond microdosimeter and the first three methods of detector material conversion whilst for the bin‐by‐bin transformation, the uncertainty is higher around 7%. It is evident that the stopping power uncertainty on the edge is dominating.

The modified microdosimetric kinetic model (MKM), proposed by Kase et al.,^33^ was adopted in this study to estimate the uncertainty propagation on the RBE_10_, where the measured microdosimetric spectra at a certain depth provides the *f*(*y*) data used in Equation (15). The saturation‐corrected dose mean lineal energy, $y^{*}$ was calculated from Equation (15), where *y*_0_ = 150 keV µm^−1^ is used to fit the cell survival data the best. For this calculation, the parameters for human salivary glands (HSG) were used where α_0_ = 0.13 Gy^−1^, β = 0.05 Gy^−2^, ρ = 1 g cm^−3^, and *r*_d_ = 0.42 µm. A correction factor was applied to convert microdosimetric measurements to water in carbon ion according to Bolst et al.^17^

(15) $$y^{*}=\frac{y_{0}^{2}\int \left(1-\exp\left(-y^{2}/y_{0}^{2}\right)\right)f\left(y\right)dy}{\int yf\left(y\right)dy}$$

(16) $$\alpha =\alpha_{0}+y^{*}\frac{\beta }{\rho \pi r_{d}^{2}}$$

where α_0_ is a constant representing the initial slope of the survival fraction curve in the limit of zero LET, β is a constant independent of LET, ρ is the density of the tissue, and *r*_d_ is the radius of the domain.

The RBE for a 10% cell survival (RBE_10_) is calculated using Equation (17), where $D_{10,R}$ is the dose required for the 10% survival irradiated by 200 keV x‐rays, and for HSG cells $D_{10,R}$ = 5 Gy.

(17) $${\mathrm{RBE}}_{10}=\frac{2\beta D_{10,R}}{\sqrt{\alpha^{2}-4\beta \ln\left(0.1\right)}-\alpha }$$

The uncertainty of the microdosimetric quantities discussed earlier in this section were propagated to estimate the contributions to the uncertainty on RBE_10_ according to the MKM model from the uncertainties on linearization, the marker point, the stopping power and the thickness (see Table 4). The overall uncertainty on RBE_10_ was found to be 2.5% in the plateau and 0.2% in R_90_. These represents only uncertainties due to the sensitivity of the MKM model on the parameters considered in the propagation and not on the uncertainty of the MKM model itself.

**TABLE 4:** RBE_10_ values according to the microdosimetric kinetic model and the contributions to their uncertainty by propagating the uncertainty of the marker point, the stopping powers, the thickness and the linearization in positions A and B as indicated in Figure 7

**Quantity**	**Depth 10.9 cm**	**Depth 15.0 cm**
RBE_10_ value	1.627	2.866
u marker point	0.001	0.0001
u stopping power	0.038	0.006
u thickness	0.004	0.0003
u linearization	0.012	0.002

The low uncertainty on RBE in R_90_ is due to the fact that, the MKM model becomes insensitive to the input data at the highest values of the lineal energy due to the saturation and this is the uncertainty of the outcome of the MKM model based on the uncertainties of ${\bar{y}}_{F}$ and not the uncertainty on RBE of the model.

### 3.3 Stopping power influence

Repeating the same methodology for the assessment of the values of ${\bar{y}}_{F}$ and ${\bar{y}}_{D}$ using SRIM stopping powers, the spectra look very similar when using either of the two stopping power tables, hence, the general behavior as function of energy is represented in similar ways in both tables. What is different is the value of the edge determined with both tables, affecting ${\bar{y}}_{F}$ and ${\bar{y}}_{D}$. In order to compare the influence of the stopping power tables, the normalization of the spectra in this section is with respect to the number of events, meaning that the diamond microdosimeter spectra were cut in a way that the number of events above the cutoff channel is the same using ICRU or SRIM tables. For the silicon spectra converted to diamond the cut was made at the same energy level.

The values of ${\bar{y}}_{F}$ and ${\bar{y}}_{D}$ are smaller using the SRIM tables, which is due to the fact that the edge in SRIM is smaller so we are shifting the spectra to lower lineal energy. For the diamond data and the first method, the difference between ICRU and SRIM is within 7%. Method 4 shows the highest difference up to 9% in the plateau between SRIM and ICRU tables, whilst methods 2 and 3 have the lowest difference around 5%. These differences are consistent with the uncertainty estimates on the stopping powers.

## 4 CONCLUSIONS

We investigated three different linearization approaches, the uncertainty of the linearization, and the edge calibration. In addition, four different methods for silicon to diamond material transformation were studied, as well as the influence of two databases of the stopping power.

It was demonstrated that the linearization method influences the microdosimetric spectra, especially for the silicon microdosimeter in the lower lineal energy by changing the position of the spectra, the difference in ${\bar{y}}_{F}$ values between the different linearization methods goes up to 17.5%. It is suggested to use the double linearization for both microdosimeters as it has the lowest uncertainty of 2% and 0.5% on ${\bar{y}}_{F}$ and ${\bar{y}}_{D}$ for the plateau and the R_90,_ respectively. The marker point calibration has a lower uncertainty around 0.1% for diamond and silicon microdosimeter, in our carbon beam as compared to literature values for proton beams.^22^ The uncertainty of the stopping power on the edge is also lower in carbon beams (5%) than for proton beams (6%–10%). Four methods to convert silicon to diamond spectra, result in significant differences. The combined uncertainty on the ${\bar{y}}_{F}$ and ${\bar{y}}_{D}$ values was 5%, while for method 4 it is 7%. The choice of stopping power tables mainly affects the values of the ${\bar{y}}_{F}$ and ${\bar{y}}_{D}$, the difference between the two databases can vary between 5% and 9% depending on the material of the detector and the detector material conversion method used. The results of this works demonstrate that the uncertainty on the stopping power data is the dominant contribution to the uncertainty on microdosimetric quantities measured both with silicon and diamond microdosimeters and to their conversions from silicon to diamond.

## CONFLICT OF INTEREST

The authors declare no conflict of interest.
